# Respiratory function monitors (RFMs) used for newborn resuscitation: accuracy and performance in the presence of leak. A bench comparison study

**DOI:** 10.1016/j.resplu.2025.101132

**Published:** 2025-10-15

**Authors:** Stephanie Morakeas, Viktoria Gruber, Murray Hinder, Thomas Drevhammar, Alistair McEwan, Mark Brian Tracy

**Affiliations:** a Neonatal Intensive Care Unit, Westmead Hospital, Sydney, Australia; b Faculty of Engineering and Information Technologies, BMET Institute, The University of Sydney, Sydney, Australia; c Medical University Graz, Department of Paediatrics and Adolescent Medicine, Division of Neonatology, Graz, Austria; d University of New South Wales, Sydney, Australia; e Department of Paediatrics and Child Health, The University of Sydney, Sydney, Australia; f Department of Women’s and Children’s Health, Karolinska Institutet, Stockholm, Sweden

**Keywords:** Newborn Resuscitation, Positive Pressure Ventilation, Respiratory Function Monitor, RFM, Leak

## Abstract

**Background:**

Use of respiratory function monitors (RFMs) during newborn resuscitation is not currently recommended by ILCOR. New-generation compact monitors have recently entered the market with portable designs offering better integration into clinical practice. Consensus on science regarding RFM use has been gained using complex legacy or research-only devices. This study aims to compare the accuracy and performance under leak conditions of two legacy RFMs with two new-generation monitors.

**Methods:**

The accuracy of new-generation RFMs (ResusRight-Juno, Monitvent-Neo100) were compared to legacy RFMs (Acutronics-Florian, Phillips-NM3) in the presence of imposed dynamic leaks (0 %, 20 %, 50 %, 90 %). Manual inflations using a Self-inflating bag (SIB) and T-piece Resuscitator (TPR) were delivered to a test lung (compliance 0.6 and 2 mL/cmH_2_O). Tidal volume, leak and pressure were measured proximal to leak by each RFM and related to traceable reference values.

**Results:**

Analysis of 1,920 inflations was performed. At zero leak, all RFMs performed with comparable accuracy for tidal volume, leak and pressure with minimal differences to reference measurements. System leak was accurately determined with tested RFMs at all levels. At 90 % system leak, RFM measured SIB-delivered volumes decreased significantly to <50 % compared with no leak; TPR-delivered volumes were less impacted and more accurate.

**Conclusion:**

The new-generation RFMs demonstrated comparable accuracy and performance to legacy devices at zero leak. All RFMs displayed accurate leak levels including 90 %. RFMs used with SIBs showed significantly reduced performance and accuracy at 90 % leak in contrast to TPR at 90% leak. New-generation RFMs simplified user interfaces may enhance benefits during clinical use.

## Highlights

• Respiratory function monitors have been shown to improve resuscitation technique.
• Legacy respiratory function monitors are complex and lack portability.
• New-generation respiratory function monitors feature a simpler and portable design.
• Accuracy of new-generation respiratory function monitors compares to legacy devices.
• Leak impacts the accuracy and performance of RFMs, but only at high leak levels.

## Introduction

The recognition and correction of mask leak during newborn resuscitation is critical to effective ventilation. Moderation of delivered volume is achieved by proxy monitoring of delivered peak inspiratory pressure (PIP) if a manometer is present, and visual assessment of chest wall rise. Unrecognised mask leak, poor response to lung compliance changes,^1^ poor device function^2^ and airway obstruction^3^ can alter the resuscitator's perception of patient response to ventilation. Under or over-delivery of tidal volume (Vt) may result in abnormal gas exchange,^4^ causing lung or brain injury.^5^, ^6^, ^7^ The use of respiratory function monitoring (RFM) during newborn resuscitation has been well published,^8^ but there is still no recommendation for its use by the International Liaison Committee on Resuscitation (ILCOR).^9^ ILCOR consensus on science^10^ states there is “insufficient evidence to recommend for or against the use of RFMs”, citing three randomised control trials.^11^, ^12^, ^13^ These three cited studies have used RFMs that are either now obsolete (Florian (Acutronics Medical Systems, Switzerland) and Philips NM3 (Philips, Netherlands) or research-only devices (Advanced Life Diagnostics). These devices are large, heavy, expensive and lacking in portability. This makes their implementation for use during resuscitation difficult and usually reserved for use by more experienced clinicians in the research context. There is a shortage of suitable respiratory monitoring devices for clinical use during newborn resuscitation. Recent technology advances in flow sensor design^14^ and computer-aided design (CAD) have seen the miniaturization of RFM function into smaller, more portable point-of-care devices that are better described as resuscitation monitors. Two new-generation resuscitation monitors studied are the Neo100 (Monivent, Sweden)^15^ and the Juno (ResusRight, Australia),^16^ which interface physically between the resuscitation device (self-inflating bag (SIB), T-piece resuscitator (TPR) or anaesthetic bag) and patient interface (facemask, laryngeal mask or endotracheal tube).

Respiratory function monitors have many differing features and complex display options (Table 1) that may be difficult for first responders to interpret in the birthing environment. Calculation of inspiratory/expiratory volume in RFMs is accomplished by integration of flow measured by an inline pneumotach. Integrator triggering can be affected by leak, dynamics of resuscitation (mask adjustment,^3^ spontaneous breathing, low inspiratory effort etc.), and the type of resuscitation device used (eg. TPR). TPR like neonatal ventilators have continuous bias flow, which adds complexity to inflation/breath detection and may introduce errors in displayed volumes and calculated leaks. Comparative studies on the performance of newborn resuscitation devices are rare and have found large variations between brands.^2^, ^26^, ^27^

**Table 1:** Tested RFM’s feature set.

	**Acutronics “Florian”** (a)	**Philips “NM3”** (b)	**ResusRight “Juno”** (c,d)	**Monivent “Neo100”** (e)
**RFM Display**				
Insp Vol (Vti)	✔	✔	**X**	**X**
Exp Vol (Vte)	✔ Breath to Breath (BtB) displayed as mL	✔ (BtB) displayed as mL	✔ (BtB) displayed as mL and baby weight icons.	✔ (BtB) displayed as mL/kg. Target range determined by entered baby weight.
Leak *	✔ (60 s avg) Leak alarm set manually with auto alarm set to max leak measurement of 50 % leak.	**X** No Leak displayed and No leak alarm.	✔ (BtB) Variable display: (No leak (0–30 % green), Some leak (30–60 % orange) and High leak (60+% red).	✔ (BtB) Variable display: Leak over 50 % displayed with LEAK value flashing in red and “reduce leak” alarm on display, rapidly flashing LED on sensor module.
PIP/PEEP	✔	✔	**X**	✔
CO2	**X**	✔	**X**	**X**
Fio2	✔ (oxygen fuel cell)	**X**	**X**	**X**
O2 Saturation	**X**	✔	**X**	**X**
Inflation rate (bpm)	✔	✔	✔	✔
Breath/ Inflation indicator	✔ (via waveform)	✔ (via waveform)	✔ (symbol)	**X** (values update on inflation, no indicator for spontaneous breath)
Respiratory (waveforms)	✔	✔	**X**	**X**
**Technical Specifications**				
Pneumotach type	Hot wire	Differential Pressure	Solid State	Differential Pressure
Dead space	1 mL	1 mL	0.9 mL	<1.5 mL
Battery operation	**X**	✔	✔	✔
Weight	3.42 kg	4.36 kg	84 g	Monitor: 1.3 kg Sensor module: 15 g
Portability	Remote mounting with cable to Pneumotach.	Remote mounting with cable to Pneumotach.	Self-contained, fits at patient interface in field of view.	Wireless sensor module at patient interface with external monitor display.
Approved for clinical use	Legacy Device	Legacy Device	Training device not yet approved for clinical use.	Approved for clinical use in Europe − CE0_402_

Source.

(a) AG AMS. *FLORIAN RESPIRATION MONITOR − Instructions for Use.*

(b) Respironics P. *NM3 Respiratory Profile Monitor with VentAssist − User Manual − Model 7900.* 2011.

(c) Sensirion. *Datasheet SFM3400-AW & SFM3400-D − Digital Flow Meter for Neonatal/Pediatric* 2022.

(d) ResusRight. JUNO Training Monitor − Instructions for Use.

(e) Monivent. *Monivent Neo100 − User Manual − N100-SY.* 2023.

* For all RFM’s leak is calculated as a percentage difference of inspiratory tidal volume [((Vti-Vte)/Vti)*100].

This bench study aimed to compare the accuracy and performance of two legacy RFM monitors (Florian and NM3) compared to two new resuscitation monitors (Juno and Neo100) in the presence of four defined leaks using different two resuscitation devices (SIB and TPR) and two lung compliances (0.6 and 2 mL/cmH_2_O). Our null hypothesis is that there will be no significant differences in accuracy and performance between RFM brands across the test combinations. To our knowledge, no prior studies have directly compared the accuracy and performance of RFMs with varying levels of reproducible system leak.

## Methods

**Fig. 1:**
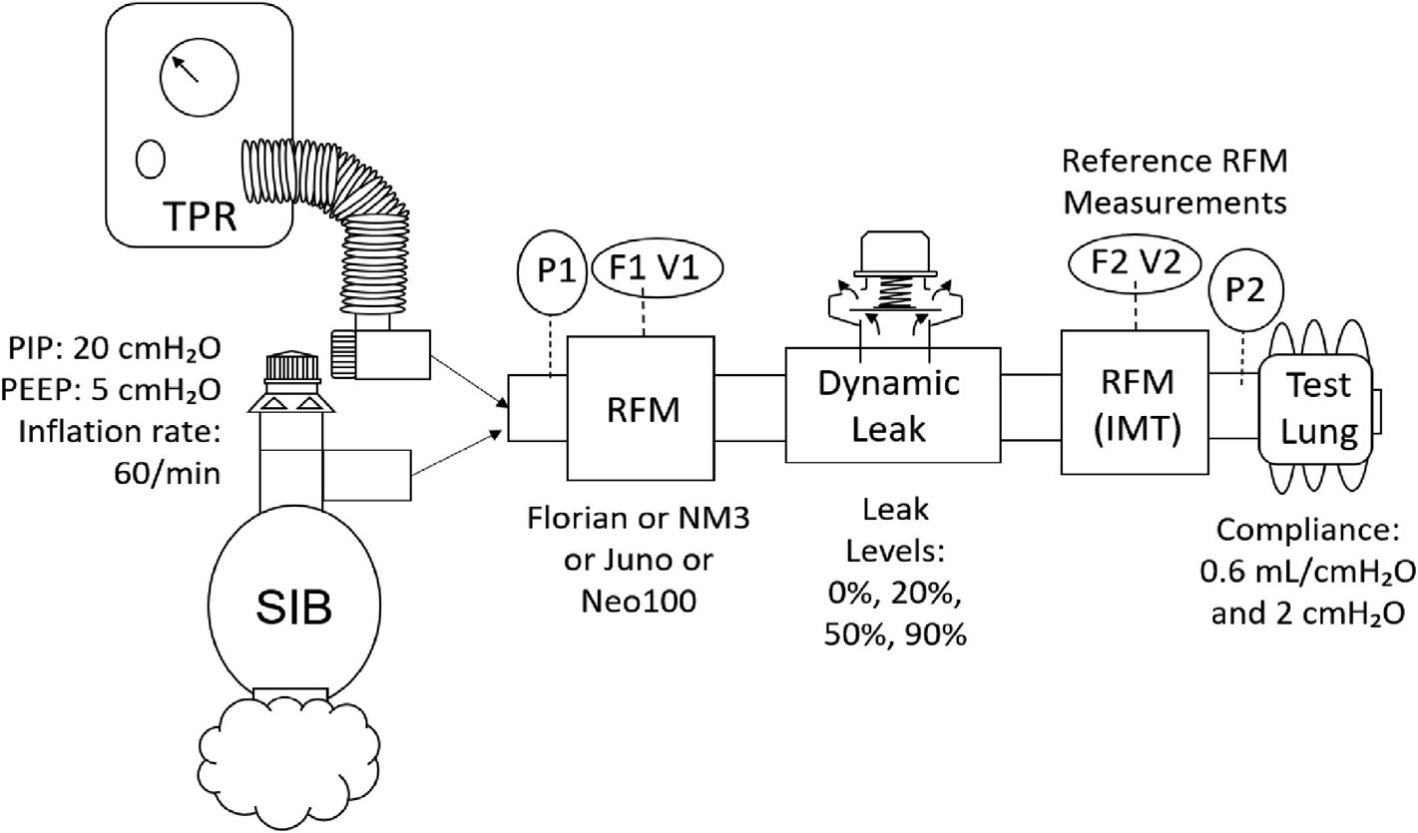
Experimental set up diagram. Note: P − Pressure, F – Flow and V – Volume measured at positions 1 and 2 by respiratory function monitors (RFMs).

This bench study investigates the accuracy and performance of legacy and novel RFMs in the presence of leak. Four RFM devices were examined in this bench study: Florian (NRM-200, SW:9B13); Respironics NM3 (Model 7900, SW:main-nm3-22); Juno (SW:V1.1.1); Neo100 (SW:V1.3.0) with two lung compliance models: 0.6 mL/cmH_2_O (Test Lung 191, Maquet, Germany) and 2 mL/cmH_2_O (Smart Lung Infant, IMT Medical, Switzerland), airway resistance 50 cmH_2_O/L/s, using both SIB (Ambu reusable Mark VI, Ambu, Denmark) with positive end expiratory pressure (PEEP) valve (Ambu disposable #199 102 001) and TPR (Neopuff, Fisher & Paykel Healthcare, New Zealand) ventilation devices. Each RFMs pneumotach was placed individually between each lung model and the manual ventilation device to measure the ventilation parameters. A traceable reference ventilation analyser (#PF-300 IMT Medical, Switzerland) (accuracy for flow and Vt of ± 1.75 % and pressure ± 0.75 %) was used to measure ventilation parameters delivered to the test lung (Fig. 1).^29^

The SIB device was robotically operated, simulating a two-finger bag squeeze previously described by Tracy et al.^2^ and the T-piece device was manually operated by a single experienced operator (cold dry air gas flow of 10 L/min), using a metronome to guide both the inflation rate and inspiratory time. The manual resuscitation devices were set up using the traceable reference device to deliver a PIP of 20 cmH_2_O, and PEEP of 5 cmH_2_O at zero leak across an inflation rate of 60/min I:E ratio 1:1.

Accuracy of RFM measurements were defined by comparing measured RFM tidal volumes to traceable reference volume and pressure measurements. This relates to the manufacturer’s pneumotach accuracy range. Performance of the RFM was defined as the ability of the RFM to present accurate data in different test conditions, such as different system leaks, mask hold methods or resuscitation devices providing PPV.

### New generation RFMs

The Neo100 is a resuscitation monitor approved for clinical use in Europe and as a resuscitation training monitor. The Neo100 features a lightweight sensor that provides colour coded guidance on delivered expiratory tidal volume (Vte) based on the patients’ weight (green: within target range, orange: above target range, red: above target range) in the patient field of view and an external monitor display with more detailed feedback on Vte (mL/kg), leak (%), pressure and ventilation rate. The device has been tested in both manikin^17^, ^18^, ^19^ and human^20^, ^21^ studies.

The Juno is a recently marketed resuscitation training monitor that is fully self-contained, portable and lightweight, with feedback displayed in the patient field of view. The Juno monitor has a display tailored to first responders with mask leak range (No leak: 0–30 % green, Some leak: 30–60 % orange, and High leak: 60+% red), Vte (displayed in mL and baby weight icons: <0.5 kg Red, 0.5–1 kg, 1–2 kg, 2–3 kg, 3–4 kg, 4–5 kg and > 5 kg Red) and inflation rate. A training self-assessment mode is incorporated, providing basic statistics on performance. The Juno has been tested in both manikin^22^, ^23^, ^24^ and human^25^ studies; the manufacturer is currently seeking regulatory approval for use as a clinical device.

### Leak model

A range of dynamic leaks was used to simulate mask leak in the system at the patient interface between the RFM pneumotach and the test lung. Our group has reported a new method of leak assessment using a dynamic leak model with pressure dependence designed to assess and compare both resuscitation devices and ventilators.^28^ This was done using a large surface area low resistance pressure relief valve (Ambu #199102001) studied in Morakeas et al.^28^ to mimic the dynamic nature of mask leak described by Gomo et al.^30^ The pressure relief valves were set and validated to the predefined leak values (0 %, 20 %, 50 % and 90 %) using the IMT analyser.^28^

### Set up and calibration

The accuracy of each RFM was determined following setup as per the manufacturer’s recommendations by comparison (pressure, flow and volume) with the IMT analyser and precision syringe (Hans Rudolph 5520 10 mL). All RFMs were found to be within each manufacturer's stated accuracy specifications. The Neo100 (Monivent) requires a patients’ weight to initiate measurements and 1 kg was used.

### Data collection and statistical analysis

Data was collected from each RFM and lung model for all combinations of ventilation device, lung compliance and leak level. Analysis was performed on the flow, volume and pressure (if applicable) waveforms to determine the RFM displayed PIP, PEEP, Vti, Vte, leak and reference RFM PIP, PEEP and Vt, 60 inflations were analysed per combination. Leak was defined as the percentage difference between Vti and Vte.

Florian respiratory data were collected and analysed using Spectra software (GroveMedical, England). Philips NM3 respiratory data were collected using the NICO Data Collection (SW:V1.5.2) and Flow Tool software (SW:V2.21.0)(Philips Respironics, Netherlands). The Juno’s and Neo100′s respiratory data was collected and calculated within the device and exported using the manufacturers research data export options. The Juno’s breath data were extracted from using ResusRight’s download software (ResusRight, Australia). The Juno device does not measure airway pressure. The Neo100′s ventilation data (breath by breath) were downloaded on a USB directly from the monitor. The reference RFM data were collected using Flowlab software (IMT Medical, Switzerland) and breath analysis was performed using LabChart8 (AdInstruments, New Zealand).

Analysis of variance (ANOVA) for repeated measures was used to determine differences in predicted means between devices, compliances and system leak levels ANOVA was reported with p value adjusted F test using Box’s conservative epsilon; p values of <0.05 we considered significant. Summary statistics including mean and standard deviation (SD) were performed to determine variance of tidal volume, leak and pressures along with their differences to reference measurements. Negative leak values between −15 % and 0 % were investigated and re-coded to 0 %. Negative leaks less than −15 % were discarded.

## Results

Analysis of 3727 inflations was performed (960 Florian, Juno, Neo100 and 847 NM3). ANOVA repeated measures analysis showed that the RFM, resuscitation device and leak level were significant in determining the Vte, leak and pressure difference between RFM and reference measurements. However, the difference (<±10 %) with imposed leak levels up to 50 % is clinically tolerable and within the device’s stated accuracy of RFMs.^28^, ^31^

### RFM accuracy

**Fig. 2:**
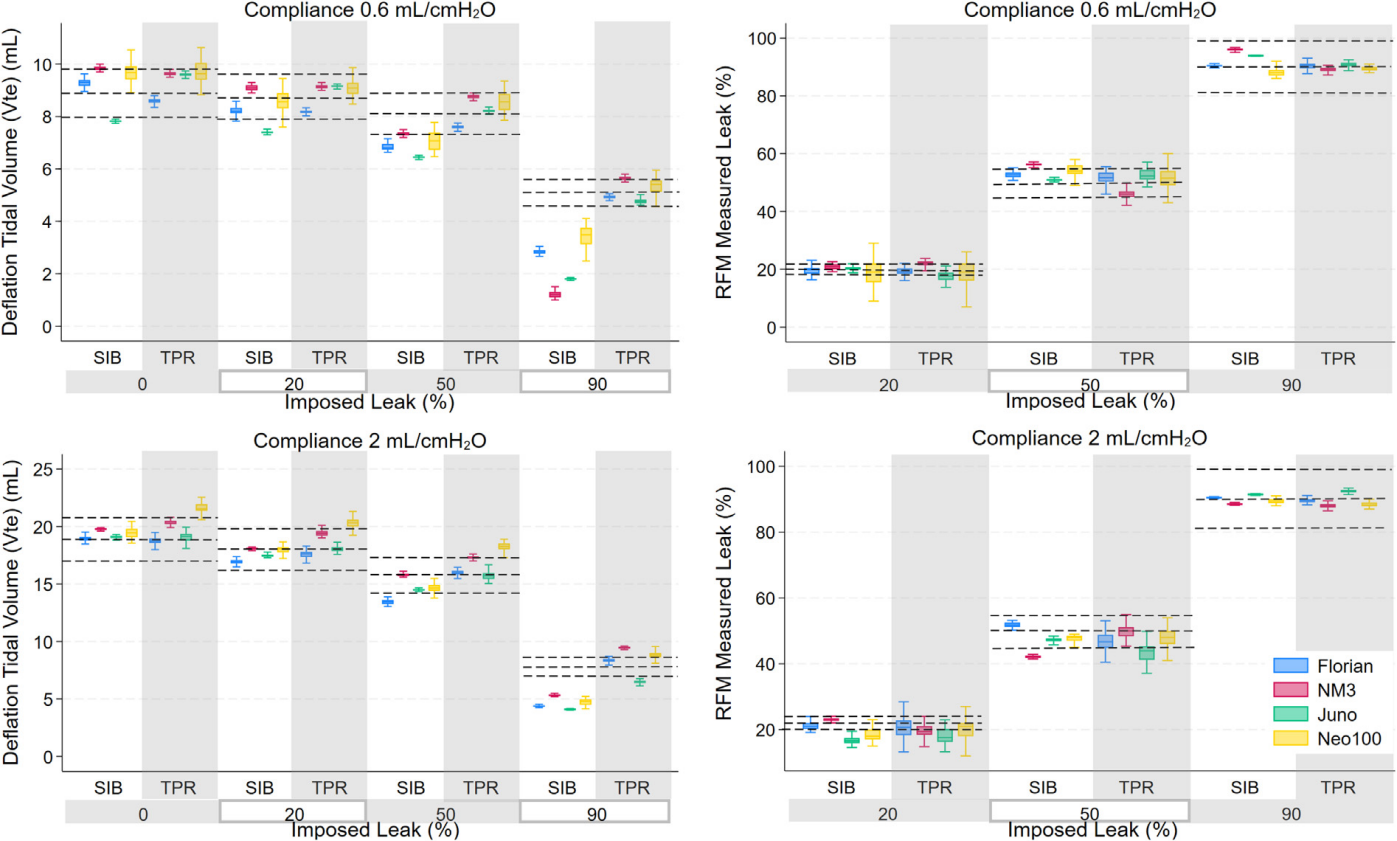
Mean deflation tidal volume and leak measured by the RFMs (Florian, NM3, Juno and Neo100) for both resuscitation devices (Self-inflating Bag (SIB) and T-piece resuscitator (TPR)), lung compliances (0.6 mL/cmH_2_O and 2 mL/cmH2O) and imposed leak levels. Reference lines represent the mean ± 10 % of reference RFM measurements for Vte and mean ± 10 % of imposed leak level for leak.

**Fig. 3:**
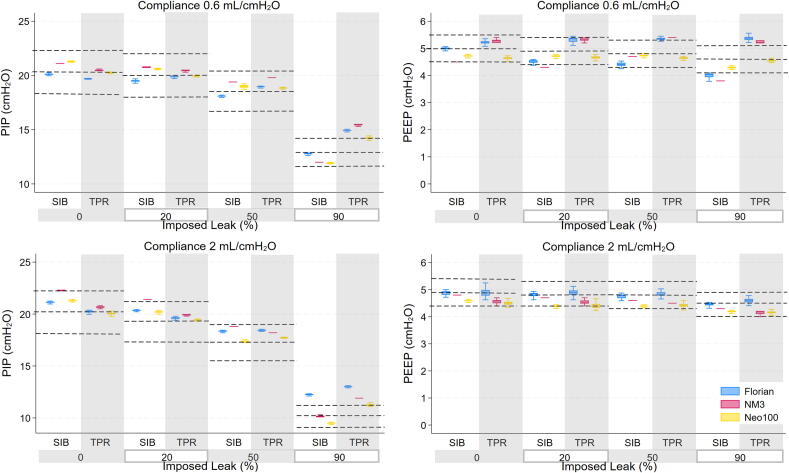
Mean PIP and PEEP measured by the RFMs (Florian, NM3, Juno and Neo100) for both resuscitation devices (Self-inflating Bag (SIB) and T-piece resuscitator (TPR)), lung compliances (0.6 mL/cmH_2_O and 2 mL/cmH2O) and imposed leak levels. Reference lines represent the mean ± 10 % of reference RFM measurements for each imposed leak level.

The performance of each RFM was variable across imposed leak levels, resuscitation device and lung compliance. All RFMs performed with a similar overall accuracy for Vte, leak and pressure measurements within ± 10 % of the reference RFM measurement with zero system leak. All RFMs showed a reduction with SIB in delivered lung volume at 90 % leak (Fig. 2, Fig. 3).

### Tidal volume

The Vte delivered decreased as leak increased, particularly at 90 % system leak. The accuracy of Vte measurements decreased as the leak increased for all RFMs, with a greater difference between the parameters measured at the RFM and reference RFM, particularly when an SIB was used (Fig. 2 & Table 2 & Supplementary Table 1 & 2). At an imposed leak of 0 % and 90 % for a lung compliance of 0.6 mL/cmH_2_O the mean differences for each RFM were Florian (SIB 0.5 mL, −1.6 mL; TPR 0.0 mL, −0.3 mL), NM3 (SIB 0.8 mL, −3.6 mL; TPR 0.6 mL, −0.2 mL), Juno (SIB −0.3 mL, −2.4 mL; TPR 0.4 mL, −1.3 mL) and Neo100 (SIB 0.3 mL, −0.9 mL; TPR 0.4 mL, −0.4 mL).

**Table 2:** Multiway table showing deflation tidal volume and leak difference between the RFM display (Florian, NM3, Juno and Neo100) and delivered test lung volume (reference RFM) as per leak (0, 20, 50, 90 %), lung compliance (Crs 0.6 and 2 cmH_2_O) and delivery device (SIB and TPR), presented as the mean ± standard deviation.

		**Leak (%) and Lung Compliance (Crs)**							
		**0 %**		**20 %**		**50 %**		**90 %**	
		**Crs 0.6**	**Crs 2**	**Crs 0.6**	**Crs 2**	**Crs 0.6**	**Crs 2**	**Crs 0.6**	**Crs 2**
**SIB**									
**Tidal Vol Difference**^a^^,^^c^**(mL)**									
	Florian	0.5 ± 0.15	0.4 ± 0.24	−0.6 ± 0.17	−0.5 ± 0.21	−1.1 ± 0.13	−1.0 ± 0.22	−1.6 ± 0.10	−2.4 ± 0.09
	NM3	0.8 ± 0.09	1.5 ± 0.10	0.1 ± 0.11	0.7 ± 0.09	−0.5 ± 0.09	0.5 ± 0.09	−3.6 ± 0.12	−1.8 ± 0.13
	Juno	−0.3 ± 0.05	0.1 ± 0.11	−0.6 ± 0.04	−0.5 ± 0.12	−1.4 ± 0.03	−0.9 ± 0.08	−2.4 ± 0.34	−3.1 ± 0.04
	Neo100	0.3 ± 0.60	0.3 ± 0.46	−0.1 ± 0.42	0.0 ± 0.34	−0.6 ± 0.37	−0.3 ± 0.31	−0.9 ± 0.45	−1.7 ± 0.30

**Leak Difference**^b^^,^^c^**(%)**									
	Florian	−0.3 ± 0.64	−0.4 ± 0.66	0.3 ± 1.79	−1.1 ± 1.07	−2.7 ± 1.17	−1.8 ± 0.80	−0.4 ± 0.32	−0.5 ± 0.19
	NM3	−4.8 ± 0.90	−4.0 ± 0.48	−0.7 ± 0.94	−3.0 ± 0.42	−6.2 ± 0.56	7.9 ± 0.39	−6.1 ± 0.39	1.5 ± 0.26
	Juno	−7.6 ± 0.83	−1.4 ± 0.89	−0.2 ± 0.74	3.4 ± 1.12	−0.9 ± 0.43	2.8 ± 0.64	−3.5 ± 1.14	−1.4 ± 0.10
	Neo100	−2.1 ± 4.54	−1.2 ± 1.53	1.0 ± 4.23	1.3 ± 1.87	−4.2 ± 2.29	2.5 ± 1.40	1.7 ± 1.47	0.6 ± 0.61

**TPR**									
**Tidal Vol Difference**^a^^,^^c^**(mL)**									
	Florian	0.0 ± 0.10	0.2 ± 0.42	−0.3 ± 0.10	−0.2 ± 0.49	−0.4 ± 0.09	−0.3 ± 0.46	−0.3 ± 0.11	−0.7 ± 0.23
	NM3	0.6 ± 0.12	1.4 ± 0.28	0.3 ± 0.09	1.0 ± 0.32	0.4 ± 0.08	0.9 ± 0.27	−0.2 ± 0.10	−0.2 ± 0.17
	Juno	0.4 ± 0.08	0.2 ± 0.71	0.2 ± 0.07	−0.4 ± 0.38	−0.3 ± 0.08	−0.8 ± 0.53	−1.3 ± 0.11	−2.1 ± 0.15
	Neo100	0.4 ± 0.44	0.5 ± 0.53	0.0 ± 0.33	0.5 ± 0.45	0.0 ± 0.37	0.4 ± 0.49	−0.4 ± 0.34	−0.4 ± 1.09

**Leak Difference**^b^^,^^c^**(%)**									
	Florian	−0.6 ± 0.78	−0.8 ± 1.14	0.8 ± 1.41	−0.6 ± 3.43	−1.8 ± 2.51	3.2 ± 3.21	−0.4 ± 1.07	0.5 ± 0.72
	NM3	−6.1 ± 1.89	−5.6 ± 1.61	−1.9 ± 0.96	0.5 ± 2.22	3.9 ± 1.73	−0.1 ± 2.27	0.9 ± 0.86	2.1 ± 0.95
	Juno	−0.2 ± 0.57	−2.5 ± 3.00	2.4 ± 1.71	2.0 ± 2.38	−2.6 ± 2.76	6.5 ± 3.08	−0.8 ± 0.99	−2.5 ± 0.47
	Neo100	−2.3 ± 3.40	−0.6 ± 1.24	1.1 ± 3.96	0.9 ± 2.61	−1.2 ± 3.33	1.1 ± 5.42	0.8 ± 0.87	−1.1 ± 0.76

a The deflation tidal volume difference is the difference between the deflation tidal volume measured by the RFM and the reference RFM (IMT) at the test lung.

b The leak difference is the difference between the leak value measured by the RFM and the set leak level.

c Tidal Volume difference, leak difference all significantly different ANOVA repeated measurements *p* < 0.001 ANOVA analysis of variance.

### Pressure

Overall, the pressure accuracy of all RFM measurements was high, particularly when a TPR was used (Fig. 2 & Supplementary Table 3 & 4). The mean PIP differences across all lung compliance and leak levels for each RFM were Florian (SIB 1.28 cmH_2_O; TPR 0.41 cmH_2_O), NM3 (SIB 1.28 cmH_2_O; TPR 0.24 cmH_2_O), Neo100 (SIB 0.81 cmH_2_O; TPR −0.18 cmH_2_O). The RFMs measured a PIP decrease as leak increased, particularly at a leak of 90 % for all combinations.

The drop in PEEP as imposed leak increased was minimal. The mean PEEP differences across lung compliance and leak levels for each RFM were Florian (SIB 0.07 cmH_2_O; TPR −0.03 cmH_2_O), NM3 (SIB −0.11 cmH_2_O; TPR −0.11 cmH_2_O), Neo100 (SIB −0.37 cmH_2_O; TPR −0.48 cmH_2_O).

## Discussion

This bench study is unique in comparing accuracy and performance of new-generation and legacy RFMs in the presence of a predefined and reproducible system leak.^28^ All RFMs operated with different pneumotach technology (Table 1) however maintained a comparable performance up to 50 % leak. The RFMs were able to measure reductions in delivered Vte and PIP as the presence of leak increases, which may be of clinical significance. As leak increased, RFMs maintained accuracy for pressure and leak measurements however, accuracy decreased with RFM-measured Vte at high leak levels (90 %). This reduced accuracy is a known limitation of RFMs, as at large leak levels, inflation/breath detection and hence Vt calculation become difficult. Although Vt accuracy is reduced, leak accuracy is maintained at 90 % leak. This finding can be attributed to changes of Vti in the presence of a dynamic leak compensating for leak flow. Increased Vti was observed with the use of a dynamic leak model.^28^ This is an important finding with significant clinical relevance. Recognition of leak with an RFM > 50 % should alert the operator that the delivered Vte’s may be lower than measured, and encourage manoeuvrers to improve the mask seal. This could involve adjustment of mask grip method, moving to a two-person 4-handed mask/PPV method,^32^ or change to a laryngeal mask airway(LMA)^33^ or endotracheal intubation.

The accuracy of legacy RFMs was investigated in a study by Verbeek et al.^34^ Both the NM3 (previous models known as Respironics CosMo) and Florian were found to have a Vt accuracy within a clinically acceptable range (<±10 %) in no-leak conditions.^34^ This result is consistent with our findings. Verbeek et al. also investigated the performance of legacy RFMs in a variety of gas conditions, changing temperature, humidity and oxygen composition, but did not investigate leak. The Florian produced a clinically relevant Vt measurement error when temperature, humidity and oxygen compensation were increased.^34^ This is attributed to the hot-wire pneumotach technology being used and should be considered in the development of RFM technology.

### RFM display vs data collection software

Most clinical studies using RFMs to guide or assess ventilation during newborn resuscitation have collected data from auxiliary software connected to RFMs. This software often provides more detailed breath-by-breath analysis of respiratory waveforms than that which is summarised on the actual screen of the RFMs. This is an important limitation and factor to consider when evaluating results of clinical and manikin studies using RFMs especially when legacy devices provide limited real-time feedback on RFM display. Particularly of note the NM3 does not display a leak percent, directly it must be calculated by the user. This may have influenced the results of clinical studies using legacy RFM devices, as clinicians respond to the information provided either on the RFM display or the display of auxiliary software tools.

### Clinical use of legacy RFMs

In 2022, two systematic reviews were published investigating the use of RFMs during neonatal resuscitation.^8^, ^35^ Both included the same three randomised control trials using legacy RFMs.^11^, ^12^, ^13^ Both concluded no significant differences in the primary outcome of hospital mortality when an RFM was used to guide PPV during newborn resuscitation. De Medeiros et al.^35^ concluded that the use of an RFM during mask ventilation reduced the incidence of brain injury and intraventricular haemorrhage. Fuerch et al.^8^ found no significant difference in severe intraventricular haemorrhage. Two out of the three randomised control trials reported a reduced portion of breaths delivered with high Vt during PPV; however it was only noted as significant in the review by De Medeiros et al.^35^ These differing conclusions highlight the need for more randomised controlled clinical trials investigating the use of RFMs to be performed to further inform ILCOR recommendations.

### Human factors and graphical user interface

Complex displays, size and screen position of legacy RFMs have been considered distracting and difficult to interpret by untrained or inexperienced resuscitators in the delivery room.^12^ Studies have been performed to evaluate human factors (e.g. eye tracking and situational awareness) during simulated neonatal resuscitation, particularly to determine if resuscitators are using or ignoring the RFM display and the impact of distraction.^36^ Distractions significantly impact the effectiveness of ventilation, reducing gaze on the RFM display, increasing mask leak and reducing minute ventilation.^37^ The use of an RFM can alter visual attention patterns of resuscitators, taking 29 % of their total gaze duration with a focus on Vte waveforms.^36^, ^38^ This shift in visual attention patterns diverts the resuscitator’s gaze away from the patient to the monitor position.^37^ However, conflicting results on the impact of gaze exist, with no effect noted using the Neopuff with gaze on pressure dial compared to focused on chest wall rise.^39^ New RFMs that provide visual indicator alarms in line with the view of the patient torso, close to the face mask, directing the resuscitator’s gaze to the patient for a longer gaze duration.^40^ Both the Juno and Neo100 only provide visual alarms, not auditory unlike legacy devices. A recent survey found visual alarms to be preferred by clinicians.^41^ A study comparing two RFM user interfaces determined that “Coloured Graphic” displays significantly reduce mask leak compared to “Flow curves” monitor displays.^42^ Although single-centre studies evaluating the use of RFMs in the NICU and delivery room showed promise, multicentre controlled trials such as the MONITOR trial failed to show benefit.^43^ The failure of studies is suggested to be linked to these very issues.^12^

### Clinical use of new-generation RFMs

In 2024, a clinical study using the Neo100 reported a statistically significant increase in the quality of ventilation delivered to patients when the monitor was used.^20^ Another simulation study demonstrated a significant reduction in mask leak with a coloured graphic (Neo100) user display.^42^ This result was also observed in a manikin study using the Juno monitor, which concluded that the use of an RFM improved face mask leak and increased the delivery of targeted ventilation.^24^ These findings contrast the results of the MONITOR trial. Although the recommendation for the use of RFMs in standard clinical practice requires further investigation, their use to support and improve resuscitation technique is a valuable training tool.^43^, ^44^

The presented study, to our knowledge, is the first to investigate the accuracy of RFMs in the presence of leak and to compare performance of devices up to 90 % system leak. It is also the first to compare the accuracy of new-generation with legacy RFMs.

### Limitations

This study has several limitations. Bench studies and findings may not be directly applicable to clinical practice. Our findings show clinically significant Vte under-estimates with SIB device at the highest imposed leak (90 %). Some difference was noted at 50 % imposed system leak, which would not be regard as clinically significant. We plan further studies within the range of 50 % and 90 % imposed system leak to answer the question of what level of system leak maintains satisfactory lung ventilation.

## Conclusions

This study demonstrated comparable accuracy and performance of new-generation (Juno and Neo100) and legacy (Florian and NM3) RFMs. Both legacy and new-generation devices show high accuracy and performance for displayed leak, Vte and pressure (if available) across all ventilation devices, compliances and leak variations, except at 90 % imposed leak, where displayed Vte error could be of clinical significance. The simpler alarms and graphic displays of new-generation RFMs show potential for adding targeted guidance during newborn resuscitation; further investigative clinical studies are required to determine optimal display parameter interface and clinical benefit of their use.

## Credit authorship contribution statement

**Stephanie Morakeas:** Writing – review & editing, Writing – original draft, Validation, Methodology, Investigation, Formal analysis, Data curation, Conceptualization. **Viktoria Gruber:** Writing – review & editing, Data curation. **Murray Hinder:** Writing – review & editing, Supervision, Methodology, Conceptualization. **Thomas Drevhammar:** Writing – review & editing, Supervision, Resources, Methodology. **Alistair McEwan:** Writing – review & editing, Supervision, Resources. **Mark Brian Tracy:** Writing – review & editing, Supervision, Formal analysis.

## Declaration of competing interest

The authors declare the following financial interests/personal relationships which may be considered as potential competing interests: ResusRight is currently a not-for-profit research organisation of which Dr Hinder and Dr Tracy are Consultants.

## References

[1] Kattwinkel J., Stewart C., Walsh B., Gurka M., Paget-Brown A. (2009). Responding to compliance changes in a lung model during manual ventilation: perhaps volume, rather than pressure, should be displayed. Pediatrics.

[2] Tracy M.B., Halliday R., Tracy S.K., Hinder M.K. (2019). Newborn self-inflating manual resuscitators: precision robotic testing of safety and reliability. Arch Dis Child Fetal Neonatal Ed.

[3] Schmolzer G.M., Dawson J.A., Kamlin C.O., O'Donnell C.P., Morley C.J., Davis P.G. (2011). Airway obstruction and gas leak during mask ventilation of preterm infants in the delivery room. Arch Dis Child Fetal Neonatal Ed.

[4] Murthy V., Dattani N., Peacock J.L. (2012). The first five inflations during resuscitation of prematurely born infants. Arch Dis Child Fetal Neonatal Ed.

[5] Barton S.K., Tolcos M., Miller S.L. (2015). Unraveling the links between the initiation of ventilation and brain injury in preterm infants. Front Pediatr.

[6] Mian Q., Cheung P.Y., O'Reilly M., Barton S.K., Polglase G.R., Schmolzer G.M. (2019). Impact of delivered tidal volume on the occurrence of intraventricular haemorrhage in preterm infants during positive pressure ventilation in the delivery room. Arch Dis Child Fetal Neonatal Ed.

[7] Pahuja A., Hunt K., Murthy V. (2018). Relationship of resuscitation, respiratory function monitoring data and outcomes in preterm infants. Eur J Pediatr.

[8] Fuerch J.H., Thio M., Halamek L.P., Liley H.G., Wyckoff M.H., Rabi Y. (2022). Respiratory function monitoring during neonatal resuscitation: a systematic review. Resusc Plus.

[9] Wyckoff M.H., Singletary E.M., Soar J. (2021). 2021 international consensus on cardiopulmonary resuscitation and emergency cardiovascular care science with treatment recommendations: summary from the basic life support; advanced life support; Neonatal life support; Education, implementation, and teams; First aid task forces; and the COVID-19 Working Group. Resuscitation.

[10] Fuerch JH RY, Thio M, Fuerch JH, et al. Respiratory Function Monitoring (NLS#806), International Liaison Committee on Resuscitation (ILCOR) Neonatal Life Support Task Force Brussels, Belgium.: ILCOR; 2022 [cited 2025 9th Jul]. Available from: https://costr.ilcor.org/document/respiratory-function-monitoring-for-neonatal-resuscitation-nls-806.

[11] Schmolzer G.M., Morley C.J., Wong C. (2012). Respiratory function monitor guidance of mask ventilation in the delivery room: a feasibility study. J Pediatr.

[12] van Zanten H.A., Kuypers K.L., van Zwet E.W. (2021). A multi-centre randomised controlled trial of respiratory function monitoring during stabilisation of very preterm infants at birth. Resuscitation.

[13] Sarrato G.Z., Luna M.S., Sarrato S.Z., Pérez A.P., Chamorro I.P., Cano J.M.B. (2019). New strategies of pulmonary protection of preterm infants in the delivery room with the respiratory function monitoring. Am J Perinatol.

[14] Sensirion_AG. Sensirion Neonatal Flow Sensor Switzerland: Sensirion AG; 2023 [cited 2025 9th Jul]. Available from: https://sensirion.com/products/catalog/SFM3400-AW/.

[15] Monivent. Monivent® Products 2024 [cited 2025 9th Jul]. Available from: https://www.monivent.se/monivent-products/monivent-neo100/.

[16] ResusRight. ResusRight “For Safer Birth” Sydney Australia2023 [cited 2025 9th Jul]. Available from: https://resusright.com/.

[17] Jenkinson A., Minamitani Y., Dassios T., Greenough A. (2024). 6633 use of a respiratory function monitor in newborn mannikin resuscitation. Arch Dis Child.

[18] Jenkinson A.C., Minamitani Y., Dassios T., Greenough A. (2024). Influence of clinical experience on newborn manikin mask ventilation performance using a respiratory function monitor. Arch Dis Child Fetal Neonatal Ed.

[19] Bibl K., Wagner M., Dvorsky R. (2025). Impact of a two-person mask ventilation technique during neonatal resuscitation: a simulation-based randomized controlled trial. J Pediatr.

[20] Dvorsky R., Bibl K., Lietz A. (2024). Optimization of manual ventilation quality using respiratory function monitoring in neonates: a two-phase intervention trial. Resuscitation.

[21] Dvorsky R., Kumer L., Leutgeb J. (2023). Optimization of ventilation strategies in preterm and term infants in a single-center intervention study. Z Geburtshilfe Neonatol.

[22] Dalley A.M., Hodgson K.A., Dawson J.A., Tracy M.B., Davis P.G., Thio M. (2024). Introducing a novel respiratory function monitor for neonatal resuscitation training. Resusc Plus.

[23] Loganathan P.K., Ashton C., Harrold E., Wigston S., Doan L.M.T., Occhipinti A. (2025). Use of real-time respiratory function monitor improves neonatal face mask ventilation: cross-over simulation study. Pediatr Anesth.

[24] Tracy M.B., Hinder M., Morakeas S. (2024). Randomised study of a new inline respiratory function monitor (Juno) to improve mask seal and delivered ventilation with neonatal manikins. Arch Dis Child Fetal Neonatal Ed.

[25] ResusRight. Feasibility study of a novel resuscitation monitoring system for measuring mask leak and tidal volumes during neonatal resuscitation. ACTRN12622000250730: Aust New Zealand Clinical Trials Registry; 2022 [cited 2025 9th Jul]. Available from: https://www.anzctr.org.au/Trial/Registration/TrialReview.aspx?id=383479&isReview=true.

[26] Hinder M., McEwan A., Drevhammer T., Donaldson S., Tracy M.B. (2019). T-piece resuscitators: how do they compare?. Arch Dis Child Fetal Neonatal Ed.

[27] Tracy M.B., Priyadarshi A., Goel D., Lowe K., Huvanandana J., Hinder M. (2017). How do different brands of size 1 laryngeal mask airway compare with face mask ventilation in a dedicated laryngeal mask airway teaching manikin?. Arch Dis Child Fetal Neonatal Ed.

[28] Morakeas S., Tracy M.B., Hinder M., Gruber V., McEwan A., Drevhammar T. (2025). Does leak matter? A novel dynamic leak model to simulate leak for performance testing of manual neonatal resuscitation devices. A bench study. Pediatr Pulmonol.

[29] IMT_Analytics_AG. Flow Analyser Manual EN 2023 [cited 2025 9th Jul]. Available from: https://imtanalytics.com/products/flowanalyser-pf-300.

[30] Gomo Ø.H., Eilevstjønn J., Holte K., Yeconia A., Kidanto H., Ersdal H.L. (2020). Delivery of positive end-expiratory pressure using self-inflating bags during newborn resuscitation is possible despite mask leak. Neonatology.

[31] Roske K., Foitzik B., Wauer R.R., Schmalisch G. (1998). Accuracy of volume measurements in mechanically ventilated newborns: a comparative study of commercial devices. J Clin Monit Comput.

[32] Tracy M.B., Klimek J., Coughtrey H. (2011). Mask leak in one-person mask ventilation compared to two-person in newborn infant manikin study. Arch Dis Child Fetal Neonatal Ed.

[33] Tracy M.B., Priyadarshi A., Goel D., Lowe K., Huvanandana J., Hinder M. (2018). How do different brands of size 1 laryngeal mask airway compare with face mask ventilation in a dedicated laryngeal mask airway teaching manikin?. Arch Dis Child Fetal Neonatal Ed.

[34] Verbeek C., van Zanten H.A., van Vonderen J.J., Kitchen M.J., Hooper S.B., Te Pas A.B. (2016). Accuracy of currently available neonatal respiratory function monitors for neonatal resuscitation. Eur J Pediatr.

[35] de Medeiros S.M., Mangat A., Polglase G.R., Sarrato G.Z., Davis P.G., Schmölzer G.M. (2022). Respiratory function monitoring to improve the outcomes following neonatal resuscitation: a systematic review and meta-analysis. Arch Dis Child Fetal Neonatal Ed.

[36] Herrick H., Weinberg D., Cecarelli C. (2020). Provider visual attention on a respiratory function monitor during neonatal resuscitation. Arch Dis Child Fetal Neonatal Ed.

[37] Bibl K., Wagner M., Dvorsky R. (2024). Impact of visual distraction on neonatal mask ventilation: a simulation-based eye-tracking study. Arch Dis Child Fetal Neonatal Ed.

[38] Katz T.A., Weinberg D.D., Fishman C.E. (2019). Visual attention on a respiratory function monitor during simulated neonatal resuscitation: an eye-tracking study. Arch Dis Child Fetal Neonatal Ed.

[39] Tracy M.B., Klimek J., Shingde V., Hinder M., Maheshwari R., Tracy S. (2010). Neopuff T‐piece mask resuscitator: is mask leak related to watching the pressure dial?. Acta Pædiatr.

[40] Ikuta Y., Takatori F., Amari S., Ito A., Ishiguro A., Isayama T. (2025). Effects of a respiratory function indicator light on visual attention and ventilation quality during neonatal resuscitation: a randomised controlled crossover simulation trial. J Perinat Med.

[41] Käferböck A.-S., Meggy H., Sieber D., Pillei M., Wald M. (2025). Device functionalities and technology acceptance for innovations in neonatal ventilation and enhanced, immediate newborn care: international, multicenter, web-based survey study. JMIR Hum Factors.

[42] Ní Chathasaigh C.M., Curley A.E., O Currain E. (2025). Comparison of two respiratory function monitors for newborn mask ventilation: a randomised crossover study using simulation. Resusc Plus.

[43] Kakkilaya V. (2024). A journey towards safe and effective neonatal resuscitation. Resuscitation.

[44] O’Currain E., Thio M., Dawson J.A., Donath S.M., Davis P.G. (2019). Respiratory monitors to teach newborn facemask ventilation: a randomised trial. Arch Dis Child Fetal Neonatal Ed.

