# Supplementary material for: Respiratory function monitors (RFMs) used for newborn resuscitation: accuracy and performance in the presence of leak. A bench comparison study
Source: Resusc Plus. 2025 Oct 15;26:101132. doi: 10.1016/j.resplu.2025.101132 (PMC12630332; doi:10.1016/j.resplu.2025.101132)
Supplement: Supplementary Data 1 [file mmc1.docx]

**Supplementary Table 1:** Multiway table showing RFM measurements of deflation tidal volume, leak and the difference between the RFM display (Florian, NM3, Juno and Neo100) and delivered test lung volume (reference RFM) as per leak (0, 20, 50, 90%), lung compliance (Crs 0.6 and 2 cmH_2_O) and delivery device (SIB), presented as the mean ± standard deviation.

| **SIB** | | **Leak (%) and Lung Compliance (Crs)** | | | | | | | | |
| --- | --- | --- | --- | --- | --- | --- | --- | --- | --- | --- |
|  |  | **0%** | | **20%** | | **50%** | | **90%** | | |
|  |  | **Crs 0.6** | **Crs 2** | **Crs 0.6** | **Crs 2** | **Crs 0.6** | **Crs 2** | **Crs 0.6** | **Crs 2** |  |
| **RFM Displayed Deflation Tidal Vol (mL)** | |  |  |  |  |  |  |  |  |  |
|  | **Florian** | 9.28 ± 0.15 | 18.95 ± 0.24 | 8.20 ± 0.17 | 16.93 ± 0.20 | 6.84 ± 0.13 | 13.45 ± 0.21 | 2.84 ± 0.09 | 4.40 ± 0.09 |  |
|  | **NM3** | 9.81 ± 0.08 | 19.80 ± 0.09 | 9.08 ± 0.09 | 18.08 ± 0.11 | 7.33 ± 0.08 | 15.84 ± 0.12 | 1.20 ± 0.12 | 5.34 ± 0.12 |  |
|  | **Juno** | 7.82 ± 0.04 | 19.10 ± 0.12 | 7.40 ± 0.05 | 17.47 ± 0.12 | 6.44 ± 0.03 | 14.5 ± 0.08 | 1.94 ± 0.34 | 4.11 ± 0.04 |  |
|  | **Neo100** | 9.56 ± 0.60 | 19.44 ± 0.48 | 8.58 ± 0.42 | 17.98 ± 0.33 | 7.06 ± 0.37 | 14.66 ± 0.36 | 3.42 ± 0.45 | 4.73 ± 0.29 |  |
| **Measured Test Lung Tidal Vol (mL)** | |  |  |  |  |  |  |  |  |  |
|  | **Florian** | 8.78 ± 0.04 | 18.58 ± 0.08 | 8.79 ± 0.05 | 17.40 ± 0.03 | 7.90 ± 0.02 | 14.42 ± 0.04 | 4.45 ± 0.05 | 6.8 ± 0.03 |  |
|  | **NM3** | 8.98 ± 0.04 | 18.31 ± 0.12 | 8.95 ± 0.06 | 17.41 ± 0.07 | 7.80 ± 0.05 | 15.36 ± 0.08 | 4.81 ± 0.03 | 7.16 ± 0.10 |  |
|  | **Juno** | 8.07 ± 0.05 | 19.02 ± 0.07 | 8.02 ± 0.04 | 18.01 ± 0.03 | 7.80 ± 0.00 | 15.36 ± 0.05 | 4.30 ± 0.00 | 7.23 ± 0.05 |  |
|  | **Neo100** | 9.22 ± 0.04 | 19.17 ± 0.08 | 8.71 ± 0.03 | 17.96 ± 0.05 | 7.70 ± 0.00 | 14.96 ± 0.14 | 4.30 ± 0.02 | 6.46 ± 0.09 |  |
| **Tidal Vol Difference^(a)^ (mL)** | |  |  |  |  |  |  |  |  |  |
|  | **Florian** | 0.50 ± 0.15 | 0.37 ± 0.24 | -0.59 ± 0.17 | -0.48 ± 0.21 | -1.05 ± 0.13 | -0.97 ± 0.22 | -1.61 ± 0.10 | -2.41 ± 0.09 |  |
|  | **NM3** | 0.83 ± 0.09 | 1.48 ± 0.10 | 0.14 ± 0.11 | 0.66 ± 0.09 | -0.47 ± 0.09 | 0.47 ± 0.09 | -3.61 ± 0.12 | -1.82 ± 0.13 |  |
|  | **Juno** | -0.25 ± 0.05 | 0.07 ± 0.11 | -0.62 ± 0.04 | -0.53 ± 0.12 | -1.36 ± 0.03 | -0.85 ± 0.08 | -2.36 ± 0.34 | -3.12 ± 0.04 |  |
|  | **Neo100** | 0.34 ± 0.60 | 0.28 ± 0.46 | -0.13 ± 0.42 | 0.02 ± 0.34 | -0.64 ± 0.37 | -0.3 ± 0.31 | -0.88 ± 0.45 | -1.72 ± 0.30 |  |
| **RFM Leak (%)** | |  |  |  |  |  |  |  |  |  |
|  | **Florian** | 0.25 ± 0.64 | 0.40 ± 0.66 | 19.66 ± 1.79 | 21.09 ± 1.07 | 52.70 ± 1.17 | 51.81 ± 0.80 | 90.42 ± 0.32 | 90.46 ± 0.19 |  |
|  | **NM3** | 4.75 ± 0.90 | 3.99 ± 0.48 | 20.7 ± 0.94 | 23.03 ± 0.42 | 56.23 ± 0.56 | 42.11 ± 0.39 | 96.07 ± 0.39 | 88.51 ± 0.26 |  |
|  | **Juno** | 7.55 ± 0.83 | 1.36 ± 0.89 | 20.24 ± 0.74 | 16.62 ± 1.12 | 50.92 ± 0.43 | 47.25 ± 0.64 | 93.49 ± 1.14 | 91.41 ± 0.10 |  |
|  | **Neo100** | 2.07 ± 4.54 | 1.23 ± 1.53 | 19.02 ± 4.23 | 18.73 ± 1.87 | 54.2 ± 2.29 | 47.55 ± 1.40 | 88.33 ± 1.47 | 89.37 ± 0.61 |  |
| **Leak Difference^(b)^ (%)** | |  |  |  |  |  |  |  |  |  |
|  | **Florian** | -0.25 ± 0.64 | -0.40 ± 0.66 | 0.34 ± 1.79 | -1.09 ± 1.07 | -2.70 ± 1.17 | -1.81 ± 0.80 | -0.42 ± 0.32 | -0.46 ± 0.19 |  |
|  | **NM3** | -4.75 ± 0.90 | -3.99 ± 0.48 | -0.70 ± 0.94 | -3.03 ± 0.42 | -6.23 ± 0.56 | 7.89 ± 0.39 | -6.07 ± 0.39 | 1.49 ± 0.26 |  |
|  | **Juno** | -7.55 ± 0.83 | -1.36 ± 0.89 | -0.24 ± 0.74 | 3.38 ± 1.12 | -0.92 ± 0.43 | 2.75 ± 0.64 | -3.49 ± 1.14 | -1.41 ± 0.10 |  |
|  | **Neo100** | -2.07 ± 4.54 | -1.23 ± 1.53 | 0.98 ± 4.23 | 1.27 ± 1.87 | -4.20 ± 2.29 | 2.45 ± 1.40 | 1.67 ± 1.47 | 0.63 ± 0.61 |  |
| ^(a)^ The deflation tidal volume difference is the difference between the deflation tidal volume measured by the RFM and the reference RFM (IMT) at the test lung.  ^(b)^ The leak difference is the difference between the leak value measured by the RFM and the set leak level. | | | | | | | | | | |

**Supplementary Table 2:** Multiway table showing RFM measurements of deflation tidal volume, leak and the difference between the RFM display (Florian, NM3, Juno and Neo100) and delivered test lung volume (reference RFM) as per Leak (0, 20, 50, 90%), lung compliance (Crs 0.6 and 2 cmH_2_O) and delivery device (TPR), presented as the mean ± standard deviation.

| **TPR** | | **Leak (%) and Lung Compliance (Crs)** | | | | | | | | | | | |
| --- | --- | --- | --- | --- | --- | --- | --- | --- | --- | --- | --- | --- | --- |
|  |  | **0%** | | | **20%** | | | **50%** | | | **90%** | | |
|  |  | **Crs 0.6** | **Crs 2** | **Crs 0.6** | | **Crs 2** | **Crs 0.6** | | **Crs 2** | **Crs 0.6** | | **Crs 2** |  |
| **RFM Displayed Deflation Tidal Vol (mL)** | |  |  |  | |  |  | |  |  | |  |  |
|  | **Florian** | 8.59 ± 0.10 | 18.76 ± 0.32 | 8.18 ± 0.07 | | 17.55 ± 0.35 | 7.61 ± 0.07 | | 15.96 ± 0.32 | 4.93 ± 0.08 | | 8.36 ± 0.19 |  |
|  | **NM3** | 9.61 ± 0.10 | 20.35 ± 0.23 | 9.12 ± 0.09 | | 19.42 ± 0.25 | 8.77 ± 0.08 | | 17.32 ± 0.19 | 5.61 ± 0.09 | | 9.47 ± 0.14 |  |
|  | **Juno** | 9.60 ± 0.07 | 19.09 ± 0.54 | 9.16 ± 0.05 | | 18.04 ± 0.25 | 8.22 ± 0.06 | | 15.72 ± 0.34 | 4.77 ± 0.10 | | 6.48 ± 0.13 |  |
|  | **Neo100** | 9.72 ± 0.44 | 20.44 ± 0.44 | 9.10 ± 0.33 | | 19.38 ± 0.39 | 8.55 ± 0.36 | | 17.25 ± 0.62 | 5.37 ± 0.32 | | 7.50 ± 0.34 |  |
| **Measured Test Lung Tidal Vol (mL)** | |  |  |  | |  |  | |  |  | |  |  |
|  | **Florian** | 8.56 ± 0.08 | 18.57 ± 0.28 | 8.51 ± 0.07 | | 17.73 ± 0.29 | 7.98 ± 0.07 | | 16.29 ± 0.27 | 5.24 ± 0.07 | | 9.05 ± 0.17 |  |
|  | **NM3** | 8.97 ± 0.07 | 18.93 ± 0.23 | 8.79 ± 0.07 | | 18.41 ± 0.24 | 8.40 ± 0.07 | | 16.48 ± 0.19 | 5.77 ± 0.05 | | 9.63 ± 0.14 |  |
|  | **Juno** | 9.20 ± 0.06 | 18.91 ± 0.42 | 9.01 ± 0.07 | | 18.40 ± 0.24 | 8.49 ± 0.07 | | 16.49 ± 0.32 | 6.06 ± 0.08 | | 8.60 ± 0.13 |  |
|  | **Neo100** | 9.35 ± 0.07 | 19.98 ± 0.29 | 9.10 ± 0.09 | | 18.88 ± 0.24 | 8.55 ± 0.07 | | 16.91 ± 0.49 | 5.75 ± 0.05 | | 7.89 ± 1.05 |  |
| **Tidal Vol Difference^(a)^ (mL)** | |  |  |  | |  |  | |  |  | |  |  |
|  | **Florian** | 0.03 ± 0.10 | 0.20 ± 0.42 | -0.34 ± 0.1 | | -0.18 ± 0.49 | -0.37 ± 0.09 | | -0.33 ± 0.46 | -0.32 ± 0.11 | | -0.69 ± 0.23 |  |
|  | **NM3** | 0.64 ± 0.12 | 1.42 ± 0.28 | 0.33 ± 0.09 | | 1.01 ± 0.32 | 0.37 ± 0.08 | | 0.85 ± 0.27 | -0.16 ± 0.1 | | -0.15 ± 0.17 |  |
|  | **Juno** | 0.40 ± 0.08 | 0.18 ± 0.71 | 0.15 ± 0.07 | | -0.36 ± 0.38 | -0.27 ± 0.08 | | -0.77 ± 0.53 | -1.29 ± 0.11 | | -2.11 ± 0.15 |  |
|  | **Neo100** | 0.37 ± 0.44 | 0.46 ± 0.53 | 0.00 ± 0.33 | | 0.51 ± 0.45 | 0.00 ± 0.37 | | 0.35 ± 0.49 | -0.39 ± 0.34 | | -0.40 ± 1.09 |  |
| **RFM Leak (%)** | |  |  |  | |  |  | |  |  | |  |  |
|  | **Florian** | 0.55 ± 0.78 | 0.77 ± 1.14 | 19.24 ± 1.41 | | 20.63 ± 3.43 | 51.80 ± 2.51 | | 46.82 ± 3.21 | 90.38 ± 1.07 | | 89.54 ± 0.72 |  |
|  | **NM3** | 6.13 ± 1.89 | 5.57 ± 1.61 | 21.85 ± 0.96 | | 19.50 ± 2.22 | 46.11 ± 1.73 | | 50.12 ± 2.27 | 89.06 ± 0.86 | | 87.88 ± 0.95 |  |
|  | **Juno** | 0.24 ± 0.57 | 2.54 ± 3.00 | 17.56 ± 1.71 | | 17.99 ± 2.38 | 52.58 ± 2.76 | | 43.47 ± 3.08 | 90.80 ± 0.99 | | 92.52 ± 0.47 |  |
|  | **Neo100** | 2.25 ± 3.40 | 0.62 ± 1.24 | 18.88 ± 3.96 | | 19.12 ± 2.61 | 51.2 ± 3.33 | | 48.95 ± 5.42 | 89.23 ± 0.87 | | 91.07 ± 0.76 |  |
| **Leak Difference^(b)^ (%)** | |  |  |  | |  |  | |  |  | |  |  |
|  | **Florian** | -0.55 ± 0.78 | -0.77 ± 1.14 | 0.76 ± 1.41 | | -0.63 ± 3.43 | -1.80 ± 2.51 | | 3.18 ± 3.21 | -0.38 ± 1.07 | | 0.46 ± 0.72 |  |
|  | **NM3** | -6.13 ± 1.89 | -5.57 ± 1.61 | -1.85 ± 0.96 | | 0.50 ± 2.22 | 3.89 ± 1.73 | | -0.12 ± 2.27 | 0.94 ± 0.86 | | 2.12 ± 0.95 |  |
|  | **Juno** | -0.24 ± 0.57 | -2.54 ± 3.00 | 2.44 ± 1.71 | | 2.01 ± 2.38 | -2.58 ± 2.76 | | 6.53 ± 3.08 | -0.80 ± 0.99 | | -2.52 ± 0.47 |  |
|  | **Neo100** | -2.25 ± 3.40 | -0.62 ± 1.24 | 1.12 ± 3.96 | | 0.88 ± 2.61 | -1.20 ± 3.33 | | 1.05 ± 5.42 | 0.77 ± 0.87 | | -1.07 ± 0.76 |  |
| ^(a)^ The deflation tidal volume difference is the difference between the deflation tidal volume measured by the RFM and the reference RFM (IMT) at the test lung.  ^(b)^ The leak difference is the difference between the leak value measured by the RFM and the set leak level. | | | | | | | | | | | | | |

**Supplementary Table 3:** Multiway table showing RFM measurements of PIP, PEEP and the difference between the RFM display (Florian, NM3, Juno and Neo100) and delivered test lung pressure (reference RFM) as per Leak (0, 20, 50, 90%), lung compliance (Crs 0.6 and 2 cmH_2_O) and delivery device (SIB), presented as the mean ± standard deviation.

| **SIB** | | **Leak (%) and Lung Compliance (Crs)** | | | | | | | | |
| --- | --- | --- | --- | --- | --- | --- | --- | --- | --- | --- |
|  |  | **0%** | | **20%** | | **50%** | | **90%** | | |
|  |  | **Crs 0.6** | **Crs 2** | **Crs 0.6** | **Crs 2** | **Crs 0.6** | **Crs 2** | **Crs 0.6** | **Crs 2** |  |
| **RFM PIP (cmH₂O)** | |  |  |  |  |  |  |  |  |  |
|  | **Test Lung (ref)** | 20.38 ± 0.45 | 20.07 ± 0.19 | 19.93 ± 0.48 | 19.08 ± 0.21 | 17.75 ± 0.15 | 16.72 ± 0.47 | 11.50 ± 0.23 | 9.48 ± 0.24 |  |
|  | **Florian** | 20.10 ± 0.07 | 21.13 ± 0.07 | 19.49 ± 0.09 | 20.33 ± 0.07 | 18.08 ± 0.09 | 18.34 ± 0.08 | 12.78 ± 0.06 | 12.24 ± 0.06 |  |
|  | **NM3** | 21.10 ± 0.02 | 22.25 ± 0.05 | 20.76 ± 0.05 | 21.39 ± 0.04 | 19.41 ± 0.06 | 18.79 ± 0.03 | 12.01 ± 0.05 | 10.13 ± 0.05 |  |
|  | **Neo100** | 21.29 ± 0.04 | 21.28 ± 0.07 | 20.60 ± 0.04 | 20.19 ± 0.08 | 18.98 ± 0.16 | 17.39 ± 0.11 | 11.91 ± 0.04 | 9.49 ± 0.06 |  |
| **PIP Difference^(a)^ (cmH₂O)** | |  |  |  |  |  |  |  |  |  |
|  | **Florian** | 0.15 ± 0.08 | 1.24 ± 0.09 | 0.18 ± 0.09 | 1.49 ± 0.08 | 0.51 ± 0.09 | 2.18 ± 0.08 | 1.61 ± 0.06 | 2.87 ± 0.07 |  |
|  | **NM3** | 1.15 ± 0.06 | 2.08 ± 0.07 | 1.04 ± 0.08 | 2.16 ± 0.07 | 1.58 ± 0.08 | 1.48 ± 0.05 | 0.23 ± 0.06 | 0.36 ± 0.05 |  |
|  | **Neo100** | 0.61 ± 0.05 | 1.35 ± 0.08 | 0.55 ± 0.05 | 1.27 ± 0.09 | 1.06 ± 0.16 | 1.01 ± 0.08 | 0.29 ± 0.05 | 0.32 ± 0.07 |  |
| **RFM PEEP (cmH₂O)** | |  |  |  |  |  |  |  |  |  |
|  | **Test Lung (ref)** | 4.89 ± 0.20 | 4.9 ± 0.10 | 4.65 ± 0.28 | 4.85 ± 0.10 | 4.54 ± 0.22 | 4.80 ± 0.08 | 4.19 ± 0.30 | 4.48 ± 0.10 |  |
|  | **Florian** | 4.98 ± 0.10 | 4.88 ± 0.08 | 4.51 ± 0.05 | 4.83 ± 0.07 | 0.07 ± 4.41 | 0.09 ± 4.76 | 3.99 ± 0.09 | 4.46 ± 0.07 |  |
|  | **NM3** | 4.50 ± 0.00 | 4.80 ± 0.02 | 4.30 ± 0.03 | 4.69 ± 0.02 | 0.02 ± 4.70 | 0.00 ± 4.60 | 3.80 ± 0.00 | 4.30 ± 0.01 |  |
|  | **Neo100** | 4.72 ± 0.03 | 4.58 ± 0.04 | 4.71 ± 0.04 | 4.39 ± 0.03 | 0.03 ± 4.73 | 0.03 ± 4.39 | 4.29 ± 0.04 | 4.19 ± 0.03 |  |
| **PEEP Difference^(b)^ (cmH₂O)** | |  |  |  |  |  |  |  |  |  |
|  | **Florian** | 0.08 ± 0.11 | 0.02 ± 0.08 | 0.16 ± 0.07 | 0.01 ± 0.08 | 0.06 ± 0.15 | 0.08 ± 0.01 | 0.09 ± 0.09 | 0.02 ± 0.07 |  |
|  | **NM3** | -0.08 ± 0.04 | -0.15 ± 0.06 | -0.12 ± 0.05 | -0.15 ± 0.03 | 0.04 ± 0.02 | 0.03 ± -0.18 | -0.09 ± 0.03 | -0.17 ± 0.03 |  |
|  | **Neo100** | -0.28 ± 0.03 | -0.43 ± 0.04 | -0.25 ± 0.04 | -0.62 ± 0.04 | 0.04 ± -0.09 | 0.03 ± -0.55 | -0.31 ± 0.04 | -0.45 ± 0.03 |  |
| ^(a)^ The PIP difference is the difference between the PIP measured by the RFM and the reference RFM (IMT) at the test lung.  ^(b)^ The PEEP difference is the difference between the PEEP measured by the RFM and the reference RFM (IMT) at the test lung. | | | | | | | | | | |

**Supplementary Table 4:** Multiway table showing RFM measurements of PIP, PEEP and the difference between the RFM display (Florian, NM3, Juno and Neo100) and delivered test lung pressure (reference RFM) as per Leak (0, 20, 50, 90%), lung compliance (Crs 0.6 and 2 cmH_2_O) and delivery device (TPR), presented as the mean ± standard deviation.

| **TPR** | | **Leak (%) and Lung Compliance (Crs)** | | | | | | | |
| --- | --- | --- | --- | --- | --- | --- | --- | --- | --- |
|  |  | **0%** | | **20%** | | **50%** | | **90%** | |
|  |  | **Crs 0.6** | **Crs 2** | **Crs 0.6** | **Crs 2** | **Crs 0.6** | **Crs 2** | **Crs 0.6** | **Crs 2** |
| **RFM PIP (cmH₂O)** | |  |  |  |  |  |  |  |  |
|  | **Test Lung (ref)** | 20.25 ± 0.38 | 20.23 ± 0.48 | 20.29 ± 0.30 | 19.48 ± 0.48 | 19.10 ± 0.35 | 17.81 ± 0.28 | 14.29 ± 0.38 | 10.84 ± 0.74 |
|  | **Florian** | 19.70 ± 0.05 | 20.23 ± 0.10 | 19.89 ± 0.06 | 19.63 ± 0.09 | 18.96 ± 0.04 | 18.42 ± 0.08 | 14.93 ± 0.07 | 13.00 ± 0.05 |
|  | **NM3** | 20.47 ± 0.06 | 20.68 ± 0.07 | 20.46 ± 0.06 | 19.92 ± 0.06 | 19.8 ± 0.02 | 18.18 ± 0.08 | 15.45 ± 0.05 | 11.89 ± 0.07 |
|  | **Neo100** | 20.26 ± 0.04 | 19.29 ± 0.12 | 19.95 ± 0.04 | 18.4 ± 0.09 | 18.84 ± 0.04 | 16.97 ± 0.03 | 14.22 ± 0.09 | 9.53 ± 0.07 |
| **PIP Difference (cmH₂O)^(a)^** | |  |  |  |  |  |  |  |  |
|  | **Florian** | 0.02 ± 0.07 | 0.15 ± 0.16 | 0.04 ± 0.08 | 0.2 ± 0.08 | 0.13 ± 0.05 | 0.41 ± 0.06 | 0.87 ± 0.07 | 1.45 ± 0.06 |
|  | **NM3** | -0.04 ± 0.10 | 0.15 ± 0.12 | 0.06 ± 0.10 | 0.15 ± 0.07 | 0.05 ± 0.04 | 0.19 ± 0.05 | 0.79 ± 0.05 | 0.59 ± 0.06 |
|  | **Neo100** | 0.04 ± 0.07 | -0.28 ± 0.16 | -0.69 ± 0.06 | -0.38 ± 0.05 | -0.03 ± 0.05 | -0.39 ± 0.04 | 0.40 ± 0.09 | -0.13 ± 0.07 |
| **RFM PEEP (cmH₂O)** | |  |  |  |  |  |  |  |  |
|  | **Test Lung (ref)** | 5.04 ± 0.23 | 4.89 ± 0.36 | 5.21 ± 0.15 | 4.83 ± 0.25 | 5.09 ± 0.28 | 4.77 ± 0.17 | 5.02 ± 0.28 | 4.43 ± 0.20 |
|  | **Florian** | 5.22 ± 0.08 | 4.89 ± 0.13 | 5.32 ± 0.10 | 4.90 ± 0.12 | 0.09 ± 5.34 | 0.13 ± 4.83 | 5.37 ± 0.08 | 4.6 ± 0.09 |
|  | **NM3** | 5.27 ± 0.05 | 4.59 ± 0.08 | 5.33 ± 0.05 | 4.51 ± 0.08 | 0.03 ± 5.40 | 0.06 ± 4.51 | 5.24 ± 0.05 | 4.15 ± 0.05 |
|  | **Neo100** | 4.63 ± 0.06 | 3.68 ± 0.07 | 4.67 ± 0.05 | 3.77 ± 0.08 | 0.04 ± 4.64 | 0.11 ± 3.85 | 4.54 ± 0.03 | 3.56 ± 0.05 |
| **PEEP Difference (cmH₂O)^(b)^** | |  |  |  |  |  |  |  |  |
|  | **Florian** | -0.02 ± 0.10 | -0.08 ± 0.17 | -0.02 ± 0.12 | -0.07 ± 0.18 | 0.10 ± 0.00 | 0.12 ± -0.06 | 0.04 ± 0.09 | 0.00 ± 0.10 |
|  | **NM3** | 0.00 ± 0.05 | -0.27 ± 0.14 | -0.01 ± 0.08 | -0.21 ± 0.13 | 0.05 ± -0.02 | 0.11 ± -0.21 | -0.02 ± 0.06 | -0.11 ± 0.08 |
|  | **Neo100** | -0.11 ± 0.07 | -0.75 ± 0.12 | -0.53 ± 0.07 | -0.76 ± 0.11 | 0.07 ± -0.12 | 0.14 ± -0.80 | -0.13 ± 0.04 | -0.66 ± 0.07 |
| ^(a)^ The PIP difference is the difference between the PIP measured by the RFM and the reference RFM (IMT) at the test lung.  ^(b)^ The PEEP difference is the difference between the PEEP measured by the RFM and the reference RFM (IMT) at the test lung. | | | | | | | | | |
